# Developing a primary care-based cognitive behavioral intervention for anxiety in children through a participatory approach: a qualitative study

**DOI:** 10.1186/s12875-026-03191-y

**Published:** 2026-01-28

**Authors:** Albin Isaksson, Johan Åhlén, Henna Hasson, Leif Eriksson

**Affiliations:** 1 Department of Global Public Health, Karolinska Institutet, SLSO, Box 45436, Stockholm, 104 31 Sweden; 2 Centre for Epidemiology and Community Medicine, Box 45436, Region Stockholm, Stockholm, 104 31 Sweden; 3 Department of Learning, Informatics, Management and Ethics, Karolinska Institutet, Stockholm, 171 77 Sweden

**Keywords:** Cognitive behavioral therapy, Primary care, Participatory research, Stepped care, Childhood anxiety

## Abstract

**Background:**

Cognitive behavioral therapy (CBT) is an effective treatment for childhood anxiety, but CBT protocols suited for primary care are lacking, which limits accessibility. This study aimed to develop a CBT intervention for children aged 7–12 years with mild to moderate anxiety suitable for Swedish primary care.

**Methods:**

A participatory design approach was used, including workshops and interviews with parents (*n* = 6), therapists (*n* = 12), managers (*n* = 3), and CBT experts (*n* = 3). Following development of a preliminary CBT protocol, children (*n* = 4) and their parents piloted the intervention. Subsequent interviews with children, parents, and therapists explored treatment experiences and informed further refinement.

**Results:**

The developed intervention, *Step by Step*, followed a stepped-care model comprising two steps: Step 1 (four group sessions and one individual session) and Step 2 (three additional individual sessions if needed). Interviews with children, parents, and therapists piloting the intervention resulted in three themes with corresponding subthemes: (a) acceptance - sense of not being alone; appreciation of interactive session activities; (b) feasibility - suitable in primary care but a therapeutic challenge to shift from group to individual format; more time in group needed (c) appropriateness - flexibility promotes relevance and fit; strategies contribute to learning and behavioral change.

**Conclusions:**

This study illustrates how an intervention can be developed to fit a specific setting. A preliminary version of the stepped-care intervention yielded feedback that will be used to finalize the protocol. Larger-scale clinical trials are planned to evaluate feasibility and effectiveness.

**Supplementary Information:**

The online version contains supplementary material available at 10.1186/s12875-026-03191-y.

## Background

Anxiety disorders are the most prevalent mental health conditions in children and are associated with significant distress and adverse effects in multiple life domains, including social functioning and academic performance [1, 2]. Beyond impairment in well-being and daily functioning, having an anxiety disorder in childhood is associated with an increased risk of developing other mental disorders and physical health issues later in life [3, 4]. Given the substantial impact, ensuring early access to effective anxiety interventions is crucial.

Cognitive behavioral therapy (CBT) is an effective treatment for anxiety disorders in children [5], yet access to CBT remains limited. The proportion of affected children who receive CBT for anxiety disorders varies across studies and populations. A British community-based survey found that less than 3% of children aged 7–11 with an anxiety disorder had received adequate CBT [6]. A Swedish register-based study reported that approximately half of the children with an anxiety disorder in primary care received CBT, but it remains unclear how many of these children received an evidence-based CBT protocol or a non-manualized intervention incorporating CBT elements [7]. Consistent with these findings, international reviews have highlighted the need to improve access to CBT for childhood anxiety [5, 8].

To improve access, broader dissemination and delivery of CBT outside specialist services are essential [9]. Primary care often represents the first point of contact for children with mental health concerns and, therefore, has the potential to play a key role in early identification and intervention [9, 10]. Delivering CBT within primary care for children with mild to moderate anxiety can provide help at an early stage, prevent symptom deterioration, and relieve pressure on specialist services. Moreover, the accessibility and continuity of primary care can facilitate family engagement and reduce stigma associated with seeking mental health support [9, 10]. However, few CBT protocols have been specifically developed and evaluated for use in primary care. Creswell and colleagues examined a brief guided parent-led CBT within British primary care. In this study, parents of children aged 5–12 years received a self-help book, four one-hour face-to-face meetings, and four phone calls with a therapist [11]. The intervention covered information about anxiety, how to challenge anxious thoughts, gradual exposure to fears, and problem-solving strategies. Following treatment, 59% of the children howed much improvement in clinician-rated anxiety severity. Another primary care trial evaluated Brief Behavioral Therapy (BBT), targeting both depression and anxiety in youth aged 7–17 years [12]. The intervention consisted of 8 to 12 sessions, involving both children and parents. The sessions focused on psychoeducation, relaxation and coping techniques, problem-solving skills, exposure training, and behavioral activation. At post-assessment, 57% of participants in te BBT group demonstrated much improvement in anxiety severity.

In the latest Cochrane review on CBT for anxiety disorders in children, only one of 86 studies was conducted in a primary care setting [5]. Most of the trials were carried out in university clinics or in specialist clinical services. Compared to specialist services, primary care mental health services are characterized by offering early and brief interventions to a heterogeneous group of patients, with less symptom severity and functional impairment. These differences in clinical practice and patient needs limit the compatibility of established CBT protocols in primary care settings [13]. As a result, primary care services and clinicians face a dilemma: they can either (a) implement evidence-based CBT protocols that are too extensive for their clinical context and exceed some of their patients’ treatment needs (for example, the group-based Cool Kids program, which involves 10 sessions of 90–120 min each [14]) or (b) make individual or local adaptations that may compromise the intervention’s validity. Both approaches pose risks to equality in healthcare. The first scenario, offering an existing evidence-based CBT protocol to a small number of patients, limits resources available for assessment and treatment of others. The second scenario, in which individual therapists and primary care services modify interventions based on local needs, resources, and preferences, may lead to inconsistencies in treatment quality and content across providers and settings. To address these issues, further development and evaluation of CBT tailored for primary care are essential to improve fit for both providers and patients [9].

When developing interventions, involving diverse stakeholders (e.g., patients, healthcare staff and their managers, researchers, and policy makers) has been recommended to enhance accuracy, fit, and sense of ownership within the specific setting [15, 16]. There has been a shift from viewing patients and healthcare staff merely as service users and providers to recognizing them as collaborators in the development process, who contribute valuable knowledge and experiences. For example, treatment accuracy can possibly be improved by asking patients about what outcomes they value and what treatment components they perceive as helpful. Similarly, to ensure a good fit within a specific healthcare setting, staff and managers can provide insights about feasible treatment formats and appropriate components. Actively involving stakeholders, particularly end users, in the development process to ensure that products or services meet their needs is known as participatory design [17]. When developing CBT interventions for youth anxiety, previous studies with participatory approaches have primarily been focused on digital interventions [18–23]. Across these studies, the number and composition of involved stakeholders have varied, from only including service users (children and/or parents) [21–23] to also involving service providers [19, 20, 24] or CBT experts [18]. Furthermore, both the nature of stakeholder involvement (e.g., defining needs, designing content, or sharing user experiences) and the continuity of participation (e.g., a single point of involvement versus engagement across multiple stages of design and production) have differed between these studies.

In intervention development, it is critical to identify core components, namely specific treatment elements that impact treatment response. However, few studies have examined CBT for pediatric anxiety at a component level. In a large clinical trial, including 488 children between 7 and 17 years, introducing exposure was associated with accelerated symptom reduction and improvement of global functioning [25]. Additionally, a dismantling study demonstrated that exposure-focused therapy was associated with better treatment outcomes compared to an intervention focusing on anxiety management strategies such as identification of anxious cognitions, problem solving, and relaxation training [26].

Regarding treatment format, there is no clear evidence that individual CBT is either more or less effective than group-based CBT for childhood anxiety [5]. Similarly, Internet-delivered CBT has shown treatment effects comparable to those of face-to-face CBT for pediatric anxiety disorders [27]. Most existing CBT interventions targeting anxiety in children involve more than 10 h of therapist contact. Nevertheless, when comparing interventions with more or less than 10 h of therapist time, there were no evident outcome differences between them [5].

Given the limited access to CBT for children and the lack of interventions specifically developed, evaluated, and aligned for primary care settings, we aimed to develop a preliminary CBT protocol for children aged 7–12 years with mild to moderate anxiety suitable for Swedish primary care. Informed by user-experience feedback from children, parents, and therapists presented in this study, the finalized protocol will be evaluated for feasibility in an upcoming pilot trial.

## Method

### Setting

The study was conducted within primary care in Stockholm, Sweden. Swedish primary care services, called The First Line Mental Health (FLMH), provide early interventions for children and adolescents with mild to moderate mental health problems (ranging from subclinical level to psychiatric disorders at a moderate level). FLMH services are characterized by a high patient flow, with most patients being self-referred, and by brief, short-term interventions. Usually, in-house clinical psychologists and social workers with basic psychotherapy training deliver interventions.

### Participants and procedure

A participatory design approach was used to develop the intervention. The level of patient and public involvement (PPI), according to the IAP2 Spectrum of Public Participation, corresponded to the *“Involve”* level [28]. Stakeholders were actively engaged at multiple stages of the development process to inform and refine the intervention, ensuring that their perspectives were incorporated, while the research team retained responsibility for final decision-making. The purpose of creating a new intervention, rather than adapting an existing CBT protocol, was to avoid being constrained by predetermined treatment components, format, and length. This flexibility allowed the intervention to better align with the needs of the primary care patients and services. Workshops and interviews were conducted with parents, children, primary care therapists, managers, and CBT experts. The goal was to combine scientific knowledge with child and parent perspectives and practical organizational knowledge. The development process followed six stages: *Stage 1*: workshop with CBT experts; *Stage 2*: workshops with FLMH therapists and managers; *Stage 3*: interviews with parents; *Stage 4*: creation of an intervention prototype, feedback from FLMH centers and revisions; *Stage 5*: testing the intervention in routine FLMH care; *Stage 6*: post-intervention interviews with children, parents and therapists. Each stage of the process is described below. Data collection began in February 2022 and was completed in January 2023.

#### Stage 1. CBT experts

A two-hour online workshop was conducted via Zoom and the online platform Padlet with three researchers specialized in CBT for childhood anxiety. They were asked about (a) what CBT components should be included in an intervention targeting children with mild to moderate anxiety and (b) recommendations regarding intervention format (including to whom the intervention should be delivered, i.e., parent and child, parent-only, etc.) and length. Since no feature of the intervention was predefined (e.g., components, format, or length), participants were not restricted to certain responses. The workshop followed an adaptive reflection methodology, a structured group technique previously used in higher education to create learning goals [29] and in intervention development [30]. The workshop involved an initial phase of individual reflection, where participants wrote down their responses on virtual sticky notes. These responses were then shared with the group, collectively categorized into meaningful themes, and discussed. No consensus was sought among participants; however, some written responses (e.g., suggestions of treatment components) were removed during the discussion phase when all participants agreed that they were redundant or irrelevant. Consequently, components were categorized as “suggested” if they (a) were mentioned (i.e., written on a sticky note) by at least one participant and (b) were not agreed upon during the discussion phase to be discarded. The second author, a PhD and clinical psychologist, moderated the workshop, while the last author, a PhD with prior experience in adaptive reflection methodology, provided technical, methodological, and data collection support.

#### Stage 2. Therapists and managers

Three separate three-hour workshops were arranged with clinical psychologists and their managers at three FLMH centers in the Stockholm region. Although no strict criteria were applied, FLMH centers with at least three therapists and located in areas with diverse socio-economic characteristics were selected. The participating FLMH centers differed in what anxiety interventions they offered. Two centers had experience providing both individual and group therapy, while one center exclusively offered individual interventions. Each workshop included four to six participants. Therapists and managers were asked which CBT components should be included in the intervention based on their experiences, and which format would be beneficial for patients and feasible within the FLMH context. The question about treatment values was asked collectively to the group and documented on a flipchart. For questions regarding intervention components and format, adaptive reflection methodology (as described in stage 1) was applied. The workshops were moderated by the first author, a PhD student and clinical psychologist, together with the second author. The last author provided methodological support and assisted with data collection.

#### Stage 3. Cool Kids parents

To inform the development of the new CBT protocol, parents were interviewed about their experiences with an existing CBT program for childhood anxiety. Individual semi-structured interviews (30–45 min) were conducted with six parents (four fathers and two mothers) who had recently participated in the group-based CBT program Cool Kids [14] at one of the FLMH centers. A convenience sampling method was used to recruit participants. The interviews were conducted by the first author, a clinical psychologist trained in CBT with previous experience of leading Cool Kids groups. The interviewer had no previous contact with the participating parents and children, apart from providing information about the study. An interview guide (see Additional File 1) was used, and all interviews took place at the FLMH center.

#### Stage 4. Creation of an intervention prototype and preliminary protocol development

Informed by the collected data in stages 1–3 and the scientific literature, an intervention prototype was developed by the first and second authors. This prototype was then presented to therapists and managers (*n* = 13) from the three FLMH centers during an online workshop, moderated by the first and second authors. Feedback on the intervention format and components was collected through a structured process. Participants first provided individual written feedback. This was followed by group discussions within each FLMH center. Finally, cross-center discussions were held between therapists and managers from all three centers. Additionally, implementation barriers and requirements were explored. Therapists were asked what they would need to deliver the intervention, while managers were asked about possibilities and barriers to integrating the intervention into their services. Based on this feedback, the intervention prototype was revised, and a preliminary protocol was developed.

#### Stage 5. Piloting the intervention in routine FLMH care

The preliminary intervention was piloted at one of the FLMH centers to generate user feedback for further refinement. Inclusion criteria were defined as (a) children aged 7–12 with (b) mild to moderate anxiety problems. Exclusion criteria were defined as having other psychiatric disorders or social issues in primary need of other interventions. After seeking help at the FLMH center (self-referral or referrals from other services, such as schools), children and their parents underwent a routine initial psychiatric assessment. A FLMH psychologist assessed the child´s symptomatology, social circumstances, and overall functioning. In line with regional primary care assessment guidelines, the Children´s Global Assessment Scale (CGAS) was used to support clinical decision-making regarding symptom severity level and appropriate level of care. A CGAS score above 60 indicates primary care level (mild to moderate severity), and scores below 50 indicate specialist care level (severe severity). Scores between 50 and 60 are within borderline range, and similar to any other CGAS score, decisions on severity level are based on an overall clinical evaluation of collected information. If eligibility criteria were met, children and parents were informed about the study and asked about participation. Four children (three girls and one boy) with a mean age of 10.3 (*SD* = 1.3), and their parents, were recruited and constituted the group. The families included in this stage had not previously participated in the Cool Kids parent interviews. The intervention was delivered by two FLMH clinical psychologists with CBT training and experience. The therapists received a written therapist manual and three hours of training in the preliminary protocol provided by the first author.

#### Stage 6. Interviews with children, parents, and therapists

Semi-structured interviews (15–30 minutes) were conducted with participating children, parents, and therapists after the intervention by the first author to explore initial acceptability, appropriateness, and feasibility of the intervention and enable further revisions (see Additional file 2 for interview guides). Interview questions were formulated to generate answers on both a general level (i.e., “How did you experience the intervention?“) and on a specific level (i.e., “Are there any strategies that you learned that have been helpful?“). Participants were interviewed individually by the first author at the FLMH center. The interviewer had no prior treatment contact with the parents or children. Participants were informed that the purpose of the interview was to explore their experiences and opinions about the intervention.

### Data collection and analysis

#### Stage 1 and 2

The workshops were audio-recorded to provide additional context for the written responses collected on sticky notes. These written responses were compiled and categorized into three predefined categories (treatment components, format, and length) to facilitate an overview of the data.

#### Stage 3

Cool Kids parent interviews were audio-recorded and transcribed verbatim. An inductive thematic analysis was conducted to identify patterns of meaning in parents’ answers. A semantic approach was applied, meaning that coding and theme development were based on the explicit content of the data. The thematic analysis followed the six-phase process described by Braun and Clarke [31, 32]. All six phases were conducted by the first author, and each phase was continually reviewed and discussed with the last author: *Familiarization with data*: the interviews were transcribed and read thoroughly; *Generating initial codes*: initial codes were generated using a data-driven approach, i.e., coding aimed to capture as many interesting aspects of data as possible without being restricted to pre-specified patterns or themes; *Searching for themes*: codes were grouped into potential themes, and an initial thematic map was drawn; *Reviewing themes*: the coded abstracts within each potential theme were checked for coherence. The potential themes were then related to the full dataset to ensure the themes represent an adequate picture of the data; *Defining and naming themes*: a written description was formulated for each theme, and subthemes were identified; *Producing the report*: the thematic analysis was reported concisely in this paper, since the parent interviews represent one of several data sources included in the study. To promote representativeness, excerpts were selected from various interview participants. To ensure confidentiality, individual participants are not presented in connection with specific excerpts in the results section. Quotes were translated from Swedish to English by the first author.

#### Stage 4

To develop the intervention prototype, data from stages 1–3 were combined with scientific literature to inform choices of intervention components and format. Individual feedback on the prototype was photographed, while feedback from group discussions was audio-recorded and transcribed verbatim. The first author compiled a written summary of the feedback, which was then reviewed and incorporated into prototype revisions by the first and second authors.

#### Stage 5–6

Interviews with children, parents, and therapists who piloted the preliminary intervention were audio-recorded and transcribed verbatim. A deductive thematic analysis was conducted, as the analysis specifically aimed to provide feedback on aspects of acceptability, appropriateness, and feasibility of the intervention. The analysis process mainly followed the six-phase process previously described in stage 3. Initial codes were identified using inductive coding, but in contrast to stage 3, the codes were sorted into the previously defined themes of acceptability, appropriateness, and feasibility. Similar to stage 3, the first author conducted all phases of the analysis, which were continually reviewed and discussed together with the last author. The analysis was guided by the definitions from Proctor and colleagues [33]: acceptability is defined as “the perception among implementation stakeholders that a given treatment, service, practice, or innovation is agreeable, palatable or satisfactory”; appropriateness is “the perceived fit, relevance or compatibility of the innovation or evidence-based practice for a given practice setting, provider, or consumer”; and feasibility is “the extent to which a new treatment or innovation can be successfully used or carried out within a given agency or setting”. In addition to provider (therapist) perspectives on feasibility, as suggested by Proctor [33], our study also incorporated consumer (children and parents) views on practicability as an aspect of feasibility in the present study.

#### Trustworthiness

To strengthen trustworthiness, we applied strategies to address credibility, transferability, dependability, and confirmability. To enhance credibility, i.e., ’the fit between participants’ views and the researcher’s representation of them [34], an iterative coding process was used in the thematic analyses, and feedback on the intervention prototype was collected from primary care therapists and managers. Transferability was sought by descriptions of participants and context, to enable the reader to judge the generalizability of the findings. Dependability, i.e., a logical and traceable research process, was maintained by documenting all steps in the data collection and analysis. To support confirmability, i.e., that the findings and interpretations are derived from the data, reflections were continuously made about how the researchers´ own experiences and expectations affect the research, i.e., reflexivity [35]. The first author, who conducted interviews and analysed data, was a clinical psychologist trained in CBT who participated in the design and production of the intervention. These factors may have affected the qualitative analyses of data. However, a preunderstanding of CBT in general, and the developed intervention specifically, is arguably advantageous when exploring others’ treatment experiences. The last author, neither with CBT training nor involvement in the design or production of the intervention, reviewed and supervised the qualitative analyses.

## Results

### Stage 1–2: input from workshops with CBT experts, therapists, and managers

#### Intervention components

Components suggested for inclusion in the intervention by each stakeholder group are presented in Table 1.

**Table 1:** Overview of suggested intervention components

Component	CBT experts	FLMH 1	FLMH 2	FLMH 3
Psychoeducation (learning about anxiety)	X	X	X	X
Exposure	X	X	X	X
Parental strategies (i.e. reinforcing behaviors that challenge anxiety and reducing overprotective behaviors)	X	X	X	X
Breathing and/or relaxation training		X	X	X
Goal setting	X	X	X	
Cognitive restructuring (identifying and challenging anxiety provoking thoughts)		X	X	X
Behavioral experiment (testing an anxious thought by doing a planned action)		X	X	X
School collaboration		X	X	X
Rewards to increase motivation			X	X
Managing stress and supporting healthy habits		X	X	
Use of valued direction (defining what is valued in life and acting in that direction)				X
Problem solving			X	
Social skills training		X		

#### Intervention format and length

During the CBT experts’ workshop, participants emphasized that decisions about intervention format depend on the needs of the patients and the healthcare context. No specific format, whether group, individual, face-to-face, or online, was pointed out as universally superior or inferior. Regarding intervention length, it was suggested that there are no evident advantages of interventions longer than eight sessions.

In workshops with FLMH centers, the most common suggestion was to deliver the intervention to both children and parents. Face-to-face intervention was the most frequently suggested format by FLMH 1 and FLMH 2. FLMH 3 also suggested face-to-face formats but additionally had ideas about mixed and user-selectable formats (including video sessions and module-based Internet-CBT). All FLMH centers requested flexibility in both length and components of the intervention based on individual needs. FLMH 1 and FLMH 3 proposed stepped-care models, starting with a brief intervention and offering an extension if needed. No FLMH center suggested an intervention longer than ten sessions. Both individual and group formats were proposed by all FLMH centers.

### Stage 3: input from Cool Kids parents

The thematic analysis of interviews with parents who had recently participated in evidence-based CBT for their children´s anxiety (the Cool Kids Program) resulted in three themes and eight subthemes (see Table 2). The themes cover both component- and format-related aspects of the treatment.

**Table 2:** Themes and subthemes from parent interviews after standard CBT (Cool Kids Program)

Theme	Subtheme
Difficulties in treatment involvement	Difficulties to motivate the children
	Workbook useful for parents, but practical activities preferable for children
	Number of sessions and time between them - a chance to practice or risk of losing intensity
The value of being a group	Support and normalization
	Split of parent and child group as facilitator
Relevant strategies with improvable usability	Parental strategies – helpful to take a step back
	Exposure – effective but sometimes hard to put into practice
	Cognitive restructuring – relevant but complex

#### Difficulties in treatment involvement

Parents reported motivational and practical obstacles to commit to and work actively with the Cool Kids Program. Most parents found it hard to motivate their children to do homework exercises, as one parent noted: “*NN had difficulty understanding why she would do those exercises if she had other homework to do.”*

Some parents also experienced difficulties in motivating their child to attend group sessions. Some expressed that it became easier to motivate their child in the latter part of the treatment when the child had experienced positive effects of the treatment. Others found it easier to motivate their children in the beginning, as the reduction of anxiety-related problems during treatment was experienced to decrease the need to work actively with the treatment strategies.

The parents generally appreciated the Cool Kids workbooks, but many reported that there was too much reading and writing for the children during the sessions. Practical exercises together and having group discussions were generally preferred over individual workbook exercises. More playful elements and hands-on practice for the children in the sessions were requested. One parent remarked: *“Maybe if you could do more practical exercises*,* instead of sitting and writing. […] to make it more playful and fun.”*

The number of sessions (nine) and the length of each session (90 min) were perceived as feasible by most parents, although there were some suggestions for both adding and reducing the number of sessions. During the latter part of the Cool Kids Program, the time between sessions increased from one to two weeks. Some found this change favorable as it gave them more time to practice strategies and do exercises at home. Others preferred weekly sessions, as more time between sessions increased demands on working independently and caused a loss of treatment intensity. One parent commented: *“You get some more time to practice stuff*,* but maybe it could have been a bit less time between some sessions. Maybe it [two weeks between sessions] makes you slack a bit as a parent.”*

#### The value of being a group

Being in a group was perceived as a beneficial part of the Cool Kids Program by all parents. Meeting others with similar difficulties and sharing experiences about the treatment was considered normalizing and supportive. One parent stated: *“I think that meeting other children that have similar problems…it was kind of a relief for him […]. He has probably felt alone with his problems.”*

The split into separate groups of parents and children during the sessions was appreciated and perceived to facilitate communication in both groups. One parent explained: *“I think it was good to split up children and parents. […] The children can relax in another way when they are together alone. And parents can talk more freely as well.”*

#### Relevant strategies with improvable usability

The components, i.e., the treatment strategies, were perceived as helpful and contributed to increased knowledge, positive changes in attitudes, and decreased anxiety-related avoidance behavior. However, some strategies were perceived as complex (cognitive restructuring) or hard to put into practice (exposure training). Learning about parental strategies was reported to increase awareness of the parents’ own emotional and behavioural reactions and how these reactions affected their children. Parents thought it was helpful to take a step back and support their children´s ability to handle anxiety-provoking thoughts and situations. One parent exemplified: *“But here we learned that it is okay to leave the children even if they were anxious when going to a birthday party or sleepover or so. To encourage them and not pick them up lightning fast.”*

Several parents found exposure was helpful, and some experienced that their children showed increased willingness and belief in confronting fears after the treatment. One parent described: *“Before it was just opposition against everything that was unknown and when someone placed demands on him […]. But now he is like: maybe I will try*,* but on a little lower level.”*

However, it was sometimes difficult for the families to get exposure done between sessions. Some experienced motivational issues, while others had difficulties coming up with what to expose for, as one parent stated: *“It felt kind of hands-on and easy to understand*,* but […] it didn’t get clear what [exposure training] to do for NN about her difficulties and worry.”*

Some parents found cognitive restructuring relevant and useful for both parents and children to manage the child’s anxious thoughts. Others experienced that the strategy as too complex for their children to understand. One parent noted: *“He didn´t understand it*,* yet. But I think it is an important part since it is much about how you relate to your thoughts.”*

### Stage 4: feedback on the intervention prototype and intervention development

The intervention prototype generally received positive feedback from the FLMH therapists and managers at the joint online workshop. Feedback on implementation barriers and requirements did not inform any revisions of the intervention. One revision was made to the prototype in response to feedback about the delivery of treatment components. The prototype initially included a session dedicated to collaboration with the child’s school. However, clinicians’ experiences of practical constraints and preferences for more flexible forms of collaboration resulted in a decision to exclude this session. It was replaced by an optional shorter session activity, where a summary could be written to the school about how they could support the child in coping with anxiety.

#### Intervention model and format

**Fig. 1:**
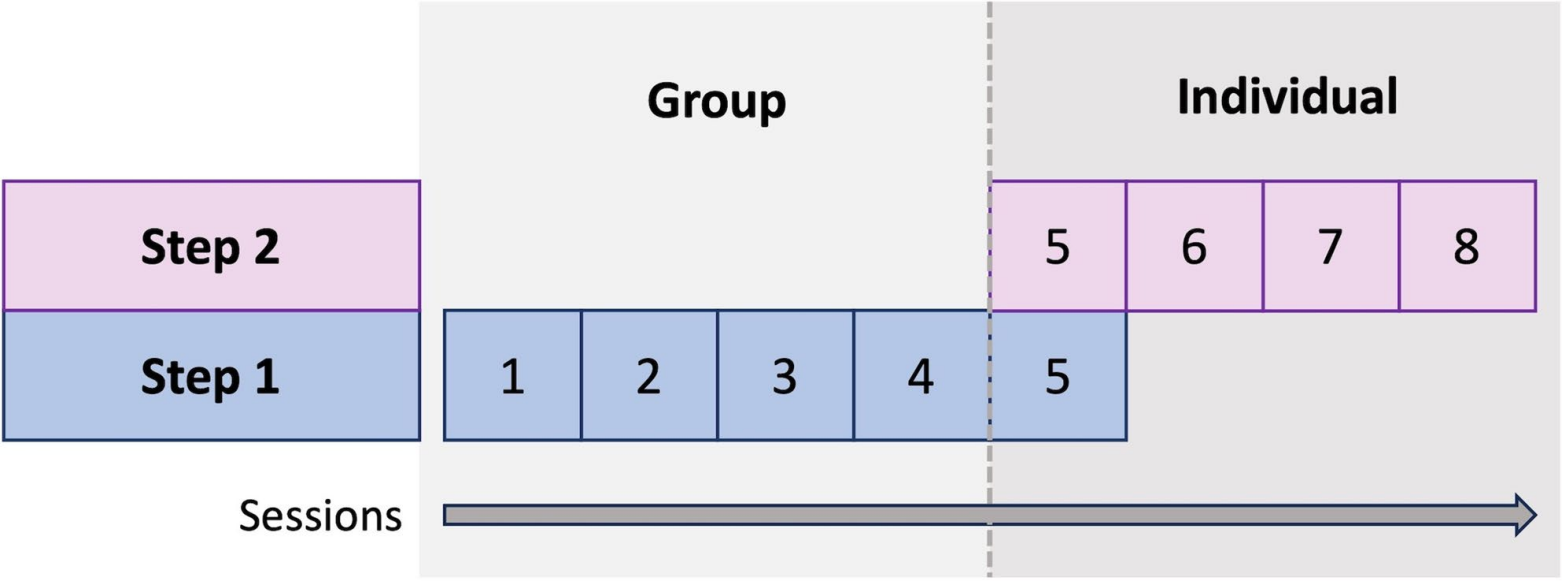
Overview of the *Step by Step* intervention

Figure 1 presents the model and format of the developed intervention, called *Step by Step*. A stepped care model was chosen based on (a) specific suggestions of a stepped-care approach from two FLMH centers (therapists and managers), and (b) general suggestions from all FLMH centers to allow flexibility in treatment length according to individual needs. The first step of the intervention, Step 1, began with four weekly group sessions (90 min each). The choice of including group sessions in Step 1 was motivated by (a) positive perceptions towards group therapy among interviewed parents in both Cool Kids and *Step by Step*, (b) suggestions from all FLMH centers, and (c) no evident superiority of individual or group therapy according to the CBT experts. Parents and children participated in three group sessions, while one session (session 2) was for parents only. Two therapists led the sessions involving both parents and children, and one therapist led the parent-only session. Including both children and parents in the sessions was suggested by all FLMH centers. Although some Cool Kids parents reported motivational issues for their children in joining group sessions, they also pointed out valuable aspects of the group format, such as normalization and support.

Parents in *Step by Step* received a textbook corresponding to the session content, including planning and registration of homework tasks. Interactive and practical session activities were emphasized in the child group, in line with suggestions from Cool Kids parent interviews about more interactive and practical session activities for children.

Session five was an individual session (45 min) to follow up treatment progress and decide together with the family whether to conclude the intervention or proceed to the second treatment step (Step 2). If continuing with step 2, the child and parent(s) were offered three additional individual sessions (45 min each). The choice of individual sessions in Step 2 was guided by requests from all FLMH centers for flexibility in treatment components to enable personalization.

The number of sessions (eight in total if completing both steps) was in line with input from CBT experts and all FLMH centers.

#### Intervention components

An overview of the components in *Step by Step* is presented in Table 3. The main component of the intervention was exposure, which was supported by all stakeholder groups (Cool Kids parents, FLMH centers, and CBT experts). As Cool Kids parents reported some difficulties in putting exposure training into practice, numerous examples of possible exposure exercises for different fears were provided, therapists supported each family in the planning of exposure training, and in-session exposure training was incorporated. The intervention also emphasized parental strategies that help parents act and respond in ways that challenge anxiety and promote their child’s autonomy. The inclusion of such strategies was supported by data from all stakeholder groups.

**Table 3:** Overview of intervention components in *Step by Step*

Session	Component
1.	Psychoeducation (learning about anxiety and how the treatment works). Goal setting. Introduction to exposure (including planning a first step to confront a fear).
2.	Parenting skills (validate emotions, reduce overprotective behaviors and encourage child autonomy). Mapping stress and vulnerability factors (healthy habits and stress level) and plan changes. Exposure (planning of gradual exposure).
3.	Exposure (doing in-session exposure and planning upcoming exposure between sessions).
4.	Exposure (follow up and planning). Parenting skills (follow up and planning). Cognitive strategies (learning about how to manage anxious thoughts). Breathing exercise.
5.	Follow-up of exposure and general treatment progress. Written information to school about how to support the child in the use of treatment strategies (if needed). *If finishing treatment*: Maintenance plan. *If continuing to step 2*: Planning of exposure. Adding of reward system to exposure if indicated. Choosing focus area.
6.	Exposure (follow-up, doing and planning). Focus area, part 1.
7.	Exposure (follow-up, doing and planning). Focus area, part 2.
8.	Follow-up: exposure and general treatment progress. Maintenance plan.

Goal setting was included based on suggestions from two FLMH centers and the CBT experts. Addressing stress and vulnerability factors was added following suggestions from two FLMH centers. Cognitive strategies were included based on input from all FLMH centers and partially positive feedback from Cool Kids parents. Since some parents described the cognitive restructuring in Cool Kids as too complex, the cognitive strategies in *Step by Step* were briefer and did not involve reading and writing. Instead, the focus was on helping children engage in actions despite anxiety-provoking thoughts, rather than attempting to modify those thoughts through cognitive reasoning.

A breathing exercise was incorporated following suggestions from all FLMH centers. The inclusion of school collaboration was likewise supported by all FLMH centers. The addition of a reward system to support exposure in Step 2, when indicated, was based on suggestions from two FLMH centers and Cool Kids parent feedback regarding children’s motivational difficulties.

Step 2 included five selectable focus areas (see Table 4) to further enhance the flexibility and personalization of the treatment, as suggested by all FLMH centers. These focus areas were designed to provide complementary and in-depth knowledge and skills, building on previously introduced components. One focus area was selected by the therapist based on the individual needs of the child and the parent(s) and was delivered in sessions six and seven.

**Table 4:** Selectable Focus Areas (Session 6 and 7)

Focus area	Description
Emotion regulation strategies	The child and parent practice emotional awareness and skills for regulating anxiety and emotions. The parents are supported in responding to the child´s emotion in a validating way.
Social skills training	The child and parent practice a social skill in a role play, which are then practiced in real life by the child.
Cognitive strategies	Anxious cognitions are identified, and cognitive strategies are learned to manage the thoughts, including behavioral experiment and cognitive restructuring. The parents are supported in responding to anxious thoughts.
Healthy habits and positive activities	Habits for sleeping, eating and physical activity are assessed and planned. Positive activities and actions for reducing stress are planned.
Parental anxiety	Parents are learned about the interplay of their own and their children´s emotional reactions. Parents are supported in responding to their own and their children's anxiety with validation and in reducing overprotective behaviors.

### Stage 5–6: initial piloting of *Step by Step*

All participating children and their parents completed the intervention. Two children finished the intervention after Step 1 (group sessions and an individual follow-up), while the other two continued with Step 2 (individual sessions). The analysis of post-intervention interviews with children, parents, and therapists resulted in six subthemes, sorted under the three deductively chosen themes acceptability, feasibility, and appropriateness (Table 5).

**Table 5:** Themes and subthemes from interviews with children, parents, and therapists

Theme	Subtheme
Acceptability	Sense of not being alone
	Appreciation of interactive session activities
Feasibility	Suitable in primary care, but a therapeutic challenge to shift from group to individual format
	More time in group needed
Appropriateness	Flexibility promotes relevance and fit
	Strategies contribute to learning and behavioral change

#### Acceptability

Children, parents, and therapists generally liked the *Step by Step* intervention and considered it a positive experience. Being together in a group and engaging in practical session activities were particularly appreciated. Most children and parents valued meeting others with similar difficulties, sharing experiences, and supporting each other. The group was reported to contribute to a sense of normalization. One of the children stated: *“I liked going to the group because it felt a bit easier when there were others with similar problems […]. It felt good not to be the only one who felt that way.”*

The practical and interactive activities in sessions were found to be fun and helpful by most children. One child noted: *“At first*,* I was nervous. But when I got here*,* it was fun […] because we did some activities. We were supposed to guess some stuff [feelings]*,* and I thought it was fun. So*,* it didn’t take a long time [to calm down]. It took about five minutes.”*

The therapists also appreciated the interactive parts of the interventions, which implied less reading and writing for the children compared to other CBT protocols. One therapist stated: “*I thought it was a lot of fun to lead the group [the children’s group]. […] Because it was so interactive*,* which I appreciated a lot with this intervention. And I think the children also liked that pretty much.”*

More hands-on training for the parent group was suggested by one of the therapists: *“Maybe if you could do some more practical […] to respond to worry…that is something you could quite easily do a role play about.”*

#### Feasibility

The intervention was viewed as feasible in the present primary care setting, but there were some difficulties for therapists when shifting from group to individual format. Activities in the intervention were considered doable from both participant and therapist perspectives, although many requested additional group session(s).

The therapists considered the intervention fit well into their services and that the processes of the intervention, from recruitment to completion, were similar to their usual way of working. As one therapist expressed it: “*We are used to offering group therapies. And it works well that some [therapists] assess [if the patient is suitable for participation in the group]*,* some therapists lead the groups and*,* if needed*,* the group leaders continue to have contact with the patients in the next phase.”*

The treatment protocol during the group sessions was perceived as manageable to follow by the therapists, but there were some difficulties adhering to the protocol in the individual sessions. One therapist noted: “*The treatment manual was so clear*,* so thoroughly worked out*,* and able to be followed. But individually it got…but I think it is always like that…it gets less smooth. And not as natural. Because you start talking about a subject*,* and then you should just “now I am going back to this [session agenda]”. It is much easier to guide the sessions in that way when being in a group. So yes*,* it was harder to stick to the agenda individually.”*

The therapists experienced that parents and children had changed expectations for the individual sessions, expecting more time to talk about topics unrelated to the intervention protocol.

Therapists, children, and parents found in-session activities and homework exercises practically doable, but many requested additional time in the first step of the treatment. One parent commented: *“It would have been beneficial to have one or two more times [in the group] to have time to do some more.”*

The therapists agreed that some more time together with the group could be beneficial to (a) strengthen group dynamics in the child group and (b) provide more time to work on treatment strategies together. One therapist reflected: *“The question is whether you need some more experiences of exposure training [in the group] […]. Possibly*,* it can be too much responsibility put on the families to manage it individually later.”*

#### Appropriateness

The stepped care model and the treatment components were reported to correspond to the diverse needs of the families and contributed to learning how to handle anxiety for children and parents. The therapists perceived the stepped care model to fit the needs of the target group. A therapist stated: “*There are a lot of patients that I would prefer to put in this treatment format. […] The families who need something more [than what was offered in the group sessions] were addressed in this treatment format. Because you can continue individually with them. So*,* I thought it fitted really well.”*

Since the participating families faced different types of issues and had varying resources, the flexibility of the treatment components was described as a key aspect. One parent pointed out: *“It felt like it wasn’t predetermined like ‘now we are doing this’. It was more like…based on the child’s own challenges and needs.”*

There was no specific treatment component considered to be missing or redundant by therapists, parents, or children, although some wanted more of certain included strategies. For example, some requested additional in-session exposure, anxiety management strategies (in addition to the breathing exercise), and parental skills to support emotional regulation in Step 1.

Several strategies in the intervention were reported to contribute to learning and behavioral change. All parents expressed that parenting skills were beneficial, including validating the child´s emotions, supporting independence, and reducing overprotective behaviors. One of the parents reflected: *I think I encourage NN a little more*,* like ‘try another time’. I don’t need to arrange everything. Maybe it feels a bit unpleasant*,* but it is not dangerous. I still think it is hard to be the one who lets her walk on her own when it is dark outside*,* or like…but I feel more confident. To not save her all the time. And that she can manage it*.”

Further, exposure training and the breathing exercise were described as helpful by children and parents. Some children described how they learned to do things they previously avoided due to anxiety, as one child noted: *“Now I can sleep in the dark.”*

Some learned how to cope with anxious feelings in situations they previously found hard to handle. One of the children noticed: *“When you breathe in and out when stress comes […]. when doing school tests […] it works better for me when doing that.”*

## Discussion

This study aimed to develop a CBT intervention for children with mild to moderate anxiety suitable for Swedish primary care. A participatory design approach was used, involving multiple stakeholders (children, parents, primary care therapists, primary care managers, and CBT experts) to inform the intervention’s development. The resulting preliminary intervention, *Step by Step*, followed a stepped-care model with two steps. Step 1 consisted of four group sessions and an individual follow-up session. If needed, the child and the parent(s) proceeded to Step 2, including three additional individual sessions. Qualitative interviews with a small group of children, parents and therapists who piloted *Step by Step* generated both positive and constructive feedback on aspects of acceptability (sense of not being alone, appreciation of interactive session activities), feasibility (suitable in primary care but a therapeutic challenge to shift from group to individual format; more time in group needed), and appropriateness (strategies contribute to learning and behavioral change).

Stepped care has been proposed as a potential way to increase access to psychological treatment for childhood anxiety [36]. There are some previous examples of stepped care CBT for children [37–40]. However, to our knowledge, this study presents the first stepped-care CBT protocol for childhood anxiety developed for use in a primary care setting. Existing stepped care interventions for childhood anxiety involved substantially more sessions compared to *Step by Step*. For example, the stepped care approach by Rapee et al. [37] involved up to 22 sessions in addition to phone sessions with parents, while the model by Van de Leden et al. [38] included 20 sessions in total. Compared to one of the few previous primary care anxiety CBT protocols [41], *Step by Step* included more therapist time and involved both children and parents, rather than delivering the intervention exclusively to parents.

Children, parents, and therapists in our study reported benefits of group-based CBT, including normalization and peer support. Similar positive peer effects have previously been described in a study of group-based parent-delivered CBT targeting children´s anxiety [42]. However, group-based CBT for broad anxiety may not be optimal for all children, particularly those with certain anxiety subtypes or comorbidities. A study on non-responders to group CBT for anxiety in youth (aged 7–17) found that those with social anxiety and comorbid mood disorders were less likely to show improvement in their primary anxiety diagnosis after treatment [43]. In qualitative interviews with these non-responding youth and their parents, youth with social phobia reported feeling nervous about what others thought of them in the group. Parents of children with a comorbid mood disorder reported that the group format limited the ability to address their family´s individual needs.

The importance of treatment flexibility in clinical practice has previously been emphasized in studies on modular CBT [44–46]. A recent review on barriers to implementing brief psychological interventions for children reported a lack of flexibility and possibilities for individualization as an obstacle to implementation [47]. In our initial qualitative evaluation of *Step by Step*, both therapists and parents valued the opportunities for individualizing the treatment. By offering individual sessions in addition to group sessions and selectable components based on individual needs, *Step by Step* can be tailored to fit a more diverse group of children.

Regarding the participant´s experiences of treatment components in *Step by Step*, the reported benefits of exposure training align with previous research [25, 26]. Parents also valued learning how to respond to the child´s anxiety, such as reducing reassurances to their children. This finding is consistent with results from a prior qualitative evaluation of a brief parent-delivered anxiety CBT [48].

Several parents and children found the breathing exercise helpful, in line with previous findings that youth prefer treatment techniques that are easy to learn, such as breathing and relaxation exercises [49]. But the inclusion of such somatic coping strategies in pediatric CBT for anxiety has been questioned [26, 50]. A meta-analysis showed that interventions incorporating somatic management strategies (including relaxation and breathing exercises) had smaller effect sizes than those that omitted such strategies [50]. In the present study, we aimed to understand experiences related to the intervention components and did not include quantitative anxiety measures. As such, a treatment component may be perceived as helpful without necessarily contributing to measurable treatment outcomes.

### Strengths and limitations

A particular strength of this study was the involvement of multiple stakeholder groups (children, parents, therapists, managers, and CBT experts) in the development of *Step by Step*. By integrating the stakeholders´ perspectives, we generated an intervention that addresses diverse needs and potentially enhances compatibility with primary care patients and providers. The involvement of stakeholders was applied throughout the development process, from generating ideas about intervention components and format to exploring experiences of the intervention in routine primary care. This continuous involvement, rather than limiting stakeholder input to the initial design phase or feasibility testing, may further enhance the fit and usefulness of the intervention.

There are some limitations to our study. Several factors may have restricted stakeholder input during the intervention´s development. Interviewed parents, who had participated in the CBT group program Cool Kids, possibly had more positive attitudes towards group therapy compared to the target group in general. These parents and their children had already agreed to participate in a group-based program. Consequently, we did not get information about the needs and preferences from the families who declined to take part in Cool Kids, introducing a risk of selection bias. The same holds for the parents and children who participated in *Step by Step*. Interviewing parents and children taking part in individual CBT may have generated other input. Further, using a convenience sampling method rather than a purposive method for the interviews with Cool Kids parents may have resulted in a sample with more positive attitudes toward the program. Potentially, the families who took part in Cool Kids but chose not to participate in the interview had other feedback about the program. Moreover, we did not collect detailed sociodemographic or diagnostic data from participants who took part in *Step by Step*, which may have restricted the scope of feedback and does limit the transferability of the findings. However, the purpose of this phase was not to evaluate a finalized protocol but to gather end-user feedback on a preliminary version of the intervention to inform final revisions. Therefore, until the protocol has been finalized and pilot testing conducted with a larger sample and more detailed participant data, it remains uncertain whether, and for whom, *Step by Step* is acceptable.

The sample sizes in both data collections of individual interviews were relatively small due to pragmatic and ethical considerations. The rationale for choosing a certain sample size in qualitative research is often based on the concept of saturation. The operationalization of saturation varies, and the use of it in thematic analysis is disputed [51]. One definition of data saturation is the point where no new information, codes, or themes are yielded from the data [52]. Possibly, larger samples may have generated new information from the interviews in the current study. Nevertheless, the purpose of this study was not an exhaustive exploration of anxiety treatment experiences, but to (a) gather information from a range of stakeholders to develop a preliminary intervention and (b) get early-phase information about feasibility aspects to inform revisions.

### Implications and future research

This study provides an example of developing an intervention through a participatory design approach, aiming to strengthen the fit and accuracy of CBT for childhood anxiety in Swedish primary care. Primary mental health care models vary considerably between countries (e.g., staff competencies, resources, and services), and we do not claim or intend that *Step by Step* should be directly feasible in other primary care contexts. Rather than developing a universal primary care intervention, we aimed to design an intervention tailored to a specific primary care setting. In this study, we provide an example of how an intervention can be developed by involving stakeholders to fit a particular context, which may be relevant for other primary care or community settings.

Larger-scale clinical trials are needed and planned to evaluate feasibility and effectiveness. The first step is a randomized pilot trial, where children in routine primary care will be randomly allocated to either the finalized *Step by Step* protocol or Cool Kids. In response to interview feedback from children, parents and therapists in the present development study, the following revisions of the intervention are considered to be needed to the finalized protocol: (a) add treatment time in the group (Step 1), (b) clarify the structure and condense the content of the individual sessions to facilitate therapists’ adherence to the manual, and (c) add interactive session activities in the parent group. In the pilot trial, detailed sociodemographic data will be collected, and the study will include an adequate sample size to evaluate feasibility, acceptability, and preliminary within-group effects. If the results are satisfactory, we intend to move on to a full-scale multi-site randomized non-inferiority trial.

## Conclusions

A stepped-care CBT intervention for childhood anxiety tailored for Swedish primary care was developed using a participatory design approach, involving multiple stakeholders. The preliminary intervention, called *Step by Step*, yielded both promising and constructive user-experience feedback regarding its acceptability, appropriateness, and feasibility, which will be used to finalize the protocol before proceeding to a pilot trial.

## Supplementary Information

Additional File 1.

Additional File 2.

## Data Availability

The datasets used and analysed during the current study are available from the corresponding author on reasonable request, subject to compliance with applicable laws and an establishment of an appropriate data agreement.

## Abbreviations

CBT: cognitive behavioral therapy
BBT: Brief Behavioral Therapy
FLMH: First Line Mental Health
PPI: Patient and Public Involvement
