# Supplementary material for: Developing a primary care-based cognitive behavioral intervention for anxiety in children through a participatory approach: a qualitative study
Source: BMC Prim Care. 2026 Jan 28;27:69. doi: 10.1186/s12875-026-03191-y (PMC12924414; doi:10.1186/s12875-026-03191-y)
Supplement: Supplementary file 1 — Additional File 1. [file 12875_2026_3191_MOESM1_ESM.pdf]

Additional file 1

Interview guide - parents about standard CBT (Cool Kids)

1. Can you tell me about your experiences of the treatment that you and your child has participated in?
   - How did you experience the group sessions?
   - How did you experience working with homework assignments in the treatment?
   - How did you perceive the workbooks?

2. a) How did you engage in the treatment?

   b) How did your child engage in the treatment?
   - What has been important to be able to engage in treatment?
   - Was there anything that made it difficult to engage in treatment?
   - Was there anything that could have increased engagement in the treatment?

3. Do you feel that the treatment has contributed to any changes for your child or for your family?

   If yes:
   - What changes?
   - In your and/or your child's behavior? Emotional changes? Ways of thinking?

   If no:
   - What do you think is the reason for that?
   - What do you think would have been needed to create a change?

4. Can you describe what in the treatment was helpful for you and/or your child?
   - In what way was it helpful for you/your child?

5. Can you describe if there were parts that were not helpful for your child?

   - What made this not helpful?

6. Are there any strategies that you learned that you and/or your child appreciated?

   - What strategies?
   - What is it that you/your child liked about those strategies?

7. Are there strategies that you learned that you and/or your child did not appreciate?

   - What strategies?
   - Why did you/your child not appreciate those strategies?

8. What do you think about the content of the treatment?

   - Do you wish there had been more or less of something in the treatment?
   - Did you feel that anything was missing in the treatment that you would like to add?

9. What do you think about the length and structure of the treatment?

   - Do you wish for any changes in the length of the treatment?
   - Do you wish for any changes in the structure of the treatment?

I have asked all my questions. Is there anything else you would like to add or ask me?
