# Supplementary material for: Developing a primary care-based cognitive behavioral intervention for anxiety in children through a participatory approach: a qualitative study
Source: BMC Prim Care. 2026 Jan 28;27:69. doi: 10.1186/s12875-026-03191-y (PMC12924414; doi:10.1186/s12875-026-03191-y)
Supplement: Supplementary file 2 — Additional File 2. [file 12875_2026_3191_MOESM2_ESM.pdf]

Additional file 2

2.1 Interview guide - children about *Step by Step*

2.2 Interview guide - parents about *Step by Step*

2.3 Interview guide - therapists about *Step by Step*

2.1 Interview guide - children about *Step by Step*

1. How do you think the treatment has been?
    - Was there anything that you liked?
    - Was there anything that you didn't like?

2. Did you get help with what you needed in the treatment?

3. Has the treatment led to any changes for you? If so, how?

Question 4 & 5: Assess whether the strategies were (1) suitable based on the child's/parent's needs (appropriateness) and/or (2) practical in application (feasibility).

4. Can you describe if there was anything in the treatment that was helpful for you?
    - Are there any things you learned (provide examples) that you found helpful?

5. Can you describe if there was anything in the treatment that was not helpful for you?
    - Are there any things you learned (provide examples) that you did NOT find helpful?

6. Was there anything missing in the treatment or something you would have liked more of?

7. How did you feel about...

    ...attending group sessions?

    ...meeting the psychologist alone with your parent(s)? (if in step 2)

    ...working on assignments at home?

8. What did you think about the duration of...

    ...the treatment? (how many times you met)

...each session? (how long each session was)

Now I have asked all my questions, is there anything else you would like to add or ask me?

2.2 Interview guide - parents about *Step by Step*

1. Can you describe your experience with the treatment that you and your child participated in?
    - Was there anything specific about the treatment that you appreciated? If yes, what?
    - Was there anything that you did not appreciate about the treatment? If yes, what?
    - Did the treatment meet your expectations? In what way?

2. Was the treatment relevant to what you/your child needed help with? In what sense?

3. Do you feel that the treatment has led to any changes for your child and/or your family? If yes, what changes?

Question 4 & 5: Assess whether the strategies were (1) suitable based on the child's/parent's needs (appropriateness) and/or (2) practical in application (feasibility).

4. Are there any strategies that you learned that have been helpful? What are they?

5. Are there strategies that you learned that have not been helpful? What are they?

6. Was there anything missing in the treatment content or something you would have liked more of?

7. What did you think about the structure of the treatment for you and your child?
    - Being in a group for the first four sessions?
    - Continuing with individual sessions? (if the child progressed to step 2)
    - Working on assignments at home?
    - Using the workbook?

8. How did you perceive the duration of the treatment?

- Number of sessions?
- Length of each session?

9. Do you have any suggestions on how the structure or duration of the treatment could be improved?

Now I have asked all my questions, is there anything else you would like to add or ask me?

2.3 Interview guide - Therapists about *Step by Step*

1. Can you describe how you experienced the treatment?
    - Was there anything about the treatment that you appreciated? If yes, what?
    - Was there anything that you did not appreciate? If yes, what?
    - Did the treatment meet your expectations? In what way?

2. How suitable and appropriate do you perceive the content of the treatment was for the participants?
    - Were there components that you found suitable/helpful? Which ones?
    - Were there components that you found unsuitable/unhelpful? Which ones?

3. Was the content feasible? In what sense?
    - Were there parts in the treatment that were difficult to do or implement?

4. Do you have any suggestions on how the content of the treatment could be improved?

5. How did you perceive the structure of the treatment?
    - Starting with four group sessions?
    - Deciding on potential continuation at session 5?
    - Continuing with individual sessions? (if the child progressed to step 2)
    - Managing participants' assignments?
    - Using the workbook?

6. How did you perceive the lenght of the treatment?
    - Number of sessions?
    - Length of each session?

7. How do you think the structure and length of the treatment fit within the framework of your primary care setting?

8. Do you have any suggestions on how the structure and/or length of the treatment could be improved?

Now I have asked all my questions, is there anything else you would like to add or ask me?
